# Multiple and Variable Binding of Pharmacologically Active Bis(maltolato)oxidovanadium(IV) to Lysozyme

**DOI:** 10.1021/acs.inorgchem.2c02690

**Published:** 2022-10-07

**Authors:** Giarita Ferraro, Maddalena Paolillo, Giuseppe Sciortino, Eugenio Garribba, Antonello Merlino

**Affiliations:** † Department of Chemical Sciences, University of Naples Federico II, Complesso Universitario di Monte Sant’Angelo, Via Cintia, I-80126Napoli, Italy; ‡ Institute of Chemical Research of Catalonia (ICIQ), The Barcelona Institute of Science and Technology, 43007Tarragona, Spain; § Dipartimento di Medicina, Chirurgia e Farmacia, Università di Sassari, Viale San Pietro, I-07100Sassari, Italy

## Abstract

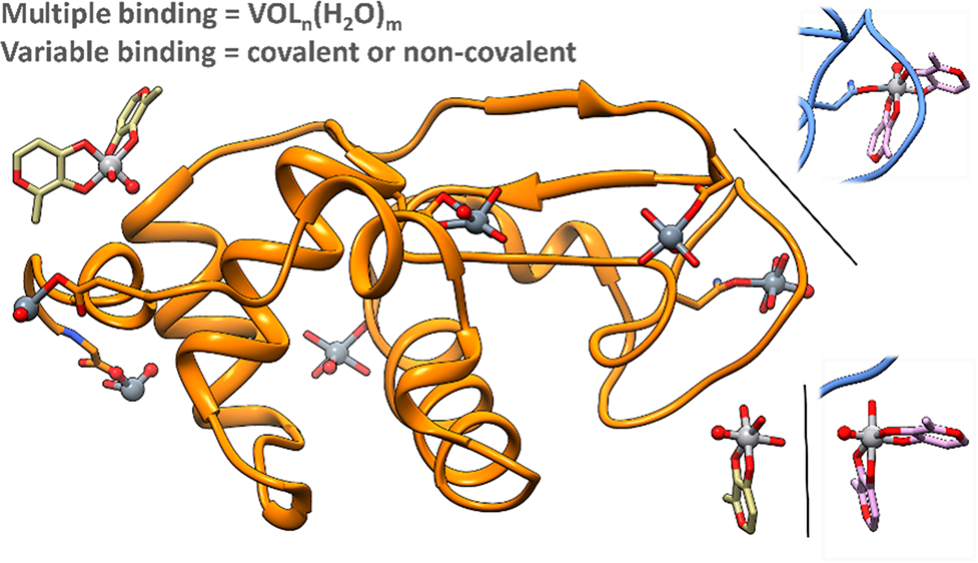

The interaction with proteins of metal-based drugs plays a crucial role in their transport, mechanism, and activity. For an active ML_*n*_ complex, where L is the organic carrier, various binding modes (covalent and non-covalent, single or multiple) may occur and several metal moieties (M, ML, ML_2_, etc.) may interact with proteins. In this study, we have evaluated the interaction of [V^IV^O(malt)_2_] (bis(maltolato)oxidovanadium(IV) or BMOV, where malt = maltolato, i.e., the common name for 3-hydroxy-2-methyl-4*H*-pyran-4-onato) with the model protein hen egg white lysozyme (HEWL) by electrospray ionization mass spectrometry, electron paramagnetic resonance, and X-ray crystallography. The multiple binding of different V-containing isomers and enantiomers to different sites of HEWL is observed. The data indicate both non-covalent binding of *cis*-[VO(malt)_2_(H_2_O)] and [VO(malt)(H_2_O)_3_]^+^ and covalent binding of [VO(H_2_O)_3–4_]^2+^ and *cis*-[VO(malt)_2_] and other V-containing fragments to the side chains of Glu35, Asp48, Asn65, Asp87, and Asp119 and to the C-terminal carboxylate. Our results suggest that the multiple and variable interactions of potential V^IV^OL_2_ drugs with proteins can help to better understand their solution chemistry and contribute to define the molecular basis of the mechanism of action of these intriguing molecules.

## Abstract 2

The interaction of [V^IV^O(malt)_2_] (BMOV, malt = maltolato) with hen egg white lysozyme (HEWL) reveals the multiple binding of different V-containing isomers and enantiomers to different sites and non-covalent and covalent binding of *cis*-[VO(malt)_2_(H_2_O)], [VO(malt)(H_2_O)_3_]^+^, [VO(H_2_O)_3−4_]^2+^, and *cis*-[VO(malt)_2_] to Glu, Asp, and Asn residues.

## Introduction

The use of metal species in diseases’ treatment is a field of extensive research. They were proposed as potential anticancer, antidiabetic, antimicrobial, antiviral, and antiarthritis drugs,^1^ with activity often higher than the organic compounds.^2^ However, rather surprisingly, they attracted less the attention of the pharmaceutical companies compared to the organic species. One of the reasons is the lack of knowledge on their transformation in the organism that is related to the interaction with bioligands and, in particular, with proteins. In fact, in bloodstream and cellular environment, for a generic pharmacologically active ML_*n*_ complex, where L is an anionic organic carrier, the binding of several metal moieties (M, ML, ML_2_, etc.) with various binding modes (covalent and non-covalent, single or multiple) may occur.^3^ This appears to be particularly important for the first-row transition metals, whose labile complexes can lose one or more ligands depending on the conditions such as pH and concentration.

In recent years, oxidovanadium(IV) complexes have attracted much attention for their medicinal potential and have been tested particularly as antidiabetic and anticancer drugs.^4^ Bis(maltolato)oxidovanadium(IV) ([V^IV^O(malt)_2_] or BMOV, where malt is maltolato or, according to the IUPAC nomenclature, 3-hydroxy-2-methyl-4*H*-pyran-4-onato), and bis(ethylmaltolato)oxidovanadium(IV) (BEOV) are among the most potent orally active insulin-mimetic agents.^5^ They have undergone extensive pre-clinical testing, and BEOV has been promoted to phase II clinical trials.^4c,5b^ Even though the experimentation as an antidiabetic drug was temporarily stopped due to renal problems of several patients and financial problems of Akesis Pharmaceuticals,^1a,6^ BMOV is usually considered the reference for the new molecules with insulin-mimetic action. Surprisingly, the tests on BMOV are continued by CFM Pharma (CFM10, Vanadis) and now it has arrived to phase II for the treatment of patients with injuries on secondary tissues caused by accidents or fire and with myocardial infarction.^1a,7^ In the solid state, [V^IV^O(malt)_2_] has a square pyramidal geometry, but when it is dissolved in water, it undergoes isomerization to the *cis*-octahedral species [V^IV^O(malt)_2_(H_2_O)],^8^ which predominates at physiological pH and shows good membrane transport properties. At a low V^IV^ concentration and/or at moderately acidic pH, the neutral complex transforms into [VO(malt)(H_2_O)_3_]^+^, while at strongly acidic pH, a significant part of V is in the free aqua ion form.^9^

Closely related to the absorption, transport, and activity of biologically active vanadium species, the binding to bioligands plays a crucial role in the development of new potential V drugs. Among the bioligands, amino acids, small peptides, and proteins have a special place. The interaction with amino acids and oligopeptides appears now rather clear and has been reviewed a few years ago,^10,11^ but much less progress has been made with proteins, both because of their intrinsic complexity and the difficulty in studying such large molecules with the instrumental and computational techniques.^12^ Up to now, several pieces of evidence suggest that the pharmacologically active vanadium complexes bind to proteins;^13,14^ the same is true for [V^IV^O(malt)_2_(H_2_O)] that interacts with several proteins and, particularly, with transferrin, albumin and immunoglobulins in blood serum,^15^ and hemoglobin in erythrocytes.^16^

To study in detail the reactivity of V complexes with proteins, small models like hen egg white lysozyme (HEWL) have been also used.^17^ It has been demonstrated through electrospray ionization mass spectrometry (ESI-MS), electron paramagnetic resonance (EPR), and computational studies (DFT, QM/MM) that potential V^IV^OL_2_ drugs react forming adducts not only with the intact V^IV^OL_2_ complex but also with the V^IV^OL^+^ and V^IV^O^2+^ ion.^12b,15h,17^ These studies revealed that V mainly coordinates to the side chains of Asp, Glu, and His residues upon replacement of water ligands or the release of one or more ligands.^12,15h^ However, experimental structural data based on X-ray diffraction (XRD) on the interaction between oxidovanadium(IV) species and proteins are still scarce.^12a^ Up to date, the following five structures were reported: V^IV^O(pic)_2_, pic = picolinato ligand, with HEWL (Asp binding),^18^ bovine pancreatic ribonuclease (RNase A, Glu binding),^19^ and trypsin (Ser binding),^20^ and, moreover, V^IV^O(bipy/phen), where bipy = 2,2′-bipyridine and phen = 1,10-phenathroline, with HEWL (simultaneous binding of Asn and Asp).^20^

To enrich the repertoire of known structures and define on structural ground the type and number of V binding sites in these biologically relevant adducts, here, we studied the [V^IV^O(malt)_2_] interaction with HEWL using ESI-MS and EPR to determine the number and type of V moieties and donors bound to protein and XRD to disclose the interacting sites and the three-dimensional structure of the metal/protein adduct.

The results can help to better understand the solution chemistry of [V^IV^O(malt)_2_], and in general of V^IV^OL_2_ potential drugs, and define the molecular basis of their transport in the organism and action mode.

## Experimental Section

### Materials

Water was deionized through the Millipore Milli-Q Academic system or purchased from Sigma-Aldrich (LC-MS grade). V^IV^OSO_4_·3H_2_O, maltol (malt), 1-methylimidazole (MeIm), 4-(2-hydroxyethyl)piperazine-1-ethanesulfonic acid (Hepes), sodium formate, sodium acetate, sodium nitrate, and ethylene glycol were Sigma-Aldrich products of the highest grade available and used as received. HEWL was purchased from Sigma-Aldrich and used without further purification. BMOV was synthesized according to the procedure reported in literature.^21^

### Spectrometric and Spectroscopic Measurements

The solutions for ESI-MS measurements were prepared dissolving in ultrapure water (LC-MS grade, Sigma-Aldrich) BMOV and HEWL to obtain a metal-to-protein molar ratio of 2/1 and a metal concentration of 100 μM. The pH of the solution was 5.0 or 6.5. ESI-MS spectra in positive-ion mode (ESI-MS(+)), immediately recorded after the solution preparation, were registered on a Q Exactive Plus Hybrid Quadrupole-Orbitrap (Thermo Fisher Scientific) mass spectrometer in the *m*/*z* range of 300–4500 with a resolution of 140000 and accumulated for at least 5 min to increase the signal-to-noise ratio. The experimental settings were a flow rate of infusion into the ESI chamber of 5.00 μL/min, spray voltage of 2300 V, capillary temperature of 250 °C, sheath gas of 10 (arbitrary units), auxiliary gas of 3 (arbitrary units), sweep gas of 0 (arbitrary units), and probe heater temperature of 50 °C. ESI-MS spectra were analyzed with Thermo Xcalibur 3.0.63 software (Thermo Fisher Scientific), and the average deconvoluted monoisotopic masses were obtained with the software Unidec 4.4.0.^22^

The EPR spectra were recorded on solutions obtained dissolving in water at pH 7.4: (i) BMOV alone, (ii) BMOV and HEWL at a molar ratio of 2/1, and (iii) BMOV and 1-methylimidazole at a ratio of 1/4. Hepes buffer (0.1 M) was also added to all the solutions. The spectra were recorded at 120 K with an X-band Bruker EMX spectrometer equipped with an HP 53150A microwave frequency counter. This was the instrumental setting: the microwave frequency was 9.40 GHz; microwave power was 20 mW; modulation frequency was 100 kHz; modulation amplitude was 4.0 G; time constant was 81.9 ms; sweep time was 335.5 s; resolution was 4096 points. To increase the signal-to-noise ratio, signal averaging was used.^13a^ The full spectra are reported in the Supporting Information, but in the text only the high-field region of the EPR spectra is shown, being more sensitive than the low-field region to the identity of the equatorial donors and amount of the several species in solution.^15a,23^ The number of scans for the high-field region of the spectra was 5.

### Crystallization

HEWL crystals were grown by using the hanging drop vapor diffusion method under two different experimental conditions: (i) 2.0 M sodium formate and 0.1 M Hepes at pH 7.5 (structures **A** and **A′**) and (ii) 20% ethylene glycol, 0.1 M sodium acetate at pH 4.0, and 0.6 M sodium nitrate (structures **B** and **B′**). These crystals were then exposed to stabilizing solutions containing the mother liquors and a saturated solution of [V^IV^O(malt)_2_] for a soaking time in the range of 3–22 days.

### Data Collection and Refinement

X-ray diffraction data were collected on four crystals of HEWL soaked with [V^IV^O(malt)_2_] (two crystals for each condition). HEWL crystals diffract X-ray in the resolution range of 1.13–1.31 Å. Data collections were carried out on Beamline XRD2 at Elettra synchrotron (Trieste, Italy), using a wavelength of 1.00 Å and a cold nitrogen stream of 100 K. Before exposure to X-ray, crystals were cryoprotected using a solution of the reservoir with 25% glycerol. Data processing and scaling were performed using a Global Phasing autoPROC pipeline.^24^ Data collection statistics are reported in the Supporting Information. Since the structures of **B** and **B′** are basically identical to each other (root mean square deviation (rmsd) 0.065 Å and the same V-containing fragments bound to the same V-binding sites), only the structure **B** is reported.

### Structure Solution and Refinement

The structures were solved by the molecular-replacement method using Phaser^25^ with PDB entries 193L(26) as templates. Refmac was used for the refinement and Coot for manual model building.^27^ The structures refine to R-factors and Rfree values within the range of 0.168–0.199 and 0.198–0.256, respectively. The V atom position was validated using anomalous difference electron density maps. Ligand positions were restraints to guide geometry optimization. The final models have good geometries and refinement statistics (see the Supporting Information). UCSF Chimera software^28^ and Pymol (www.pymol.org) were used to generate molecular graphic figures. During refinement, solvent molecules were added to the model when they had reasonable electron density levels in the 2Fo-Fc and Fo-Fc maps and were within hydrogen bonding distances to possible donors or acceptors. Coordinates and structure factors of the adduct were deposited in the Protein Data Bank^29^ under the accession codes 8AJ3, 8AJ4, and 8AJ5.

## Results and Discussion

### Behavior of BMOV in Aqueous Solution

**Figure 1:**
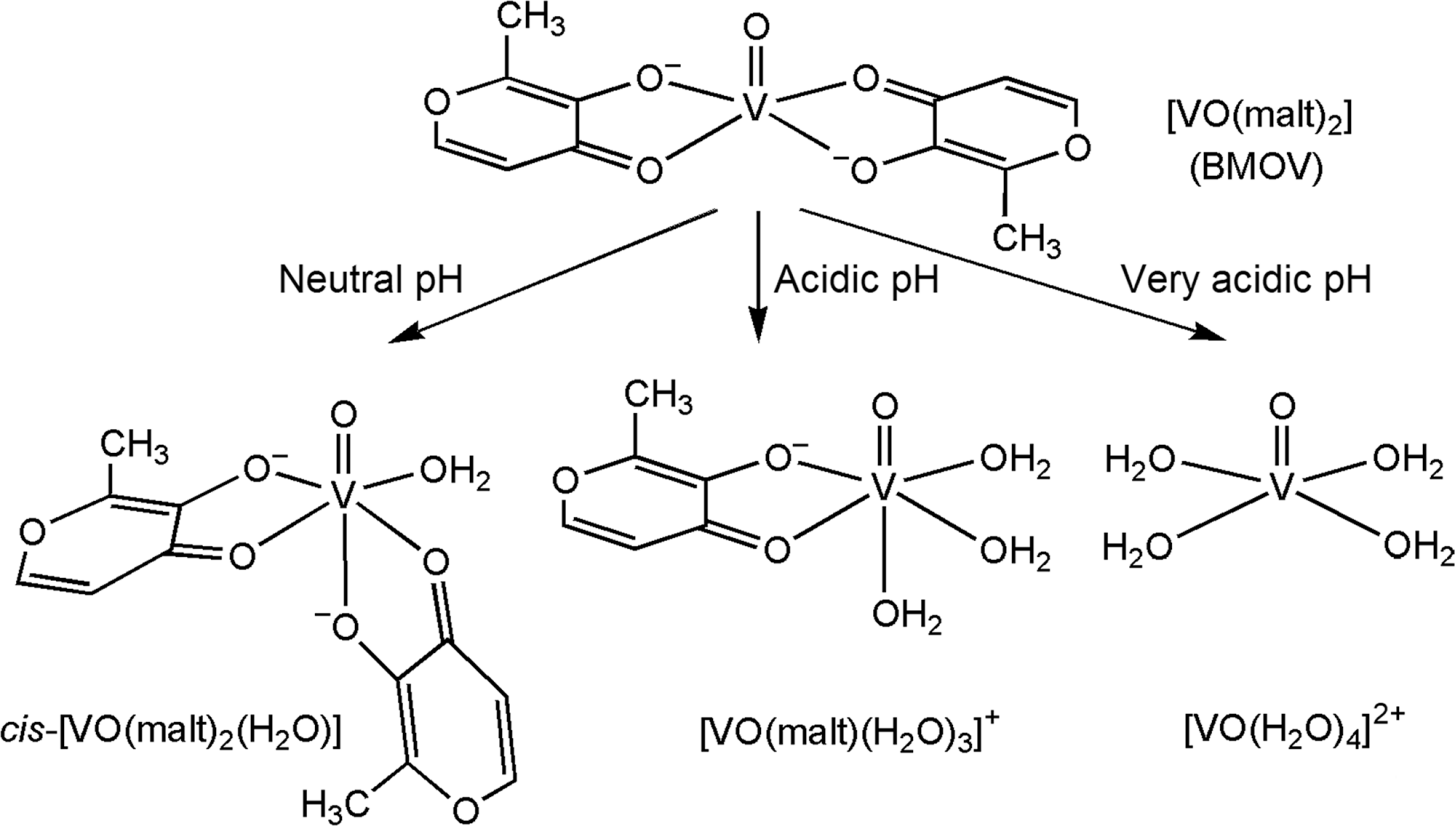
Structure of [V^IV^O(malt)_2_] (BMOV) and its possible transformations in aqueous solution. The shown behavior corresponds to a V^IV^ concentration around 1 mM.

Maltol is a naturally occurring compound able to form chelate complexes with hard metal ions through the 3-hydroxy-4-pyrone moiety. In water, it can undergo the deprotonation to maltolato (malt^–^) with a p*K_a_* of 8.44.^9^ The structure of the solid complex formed by malt^–^ with the V^IV^O^2+^ ion has been solved by XRD analysis and has a stoichiometry [V^IV^O(malt)_2_] (BMOV) with a square pyramidal geometry and two anionic ligands coordinated in the equatorial position (Figure 1).^8a^

Once BMOV is dissolved in water, the square pyramidal → *cis*-octahedral isomerization of [V^IV^O(malt)_2_] to *cis*-[V^IV^O(malt)_2_(H_2_O)] (*SPY*-5 → *OC*-6) occurs.^8b,9,30^ It must be noted that, when *OC*-6 is formed, all the eight possible isomers in equilibrium to each other exist: *OC*-6-34-Δ, *OC*-6-34-Λ, *OC*-6-24-Δ, *OC*-6-24-Λ, *OC*-6-32-Δ, *OC*-6-32-Λ, *OC*-6-23-Δ, and *OC*-6-23-Λ (Figure S1). Moreover, depending on pH, the aqua ion [V^IV^O(H_2_O)_4_]^2+^ (very acidic values) and the complexes [V^IV^O(malt)(H_2_O)_3_]^+^ (acidic values) and [V^IV^O(malt)_2_(H_2_O)] (neutral values) are formed (Figure 1).^9^ In principle, all these species could react with a protein.

In aqueous solution, the complexation is influenced not only by the pH but also by the vanadium concentration. When it is 1 mM, the 1:1 species exists between pH 3 and 4, the 1:2 complex *cis*-[V^IV^O(malt)_2_(H_2_O)] predominates between 4.5 and 8.5 and, subsequently, transforms to [V^IV^O(malt)_2_(OH)]^−^ with a p*K* of 8.79 upon the deprotonation of the equatorial water ligand (the concentration distribution curves are shown in Figure S2A);^9^ with lowering the V concentration to 100 μM, the hydrolysis becomes important: for [V^IV^O(malt)(H_2_O)_3_]^+^, the maximum concentration shifts around pH 4.5, the pH range of existence of *cis*-[V^IV^O(malt)_2_(H_2_O)] narrows, and [(V^IV^O)_2_(OH)_5_]^−^ becomes the major species in solution at pH higher than 8 (Figure S2B).^9^

### ESI-MS and EPR Studies

**Figure 2:**
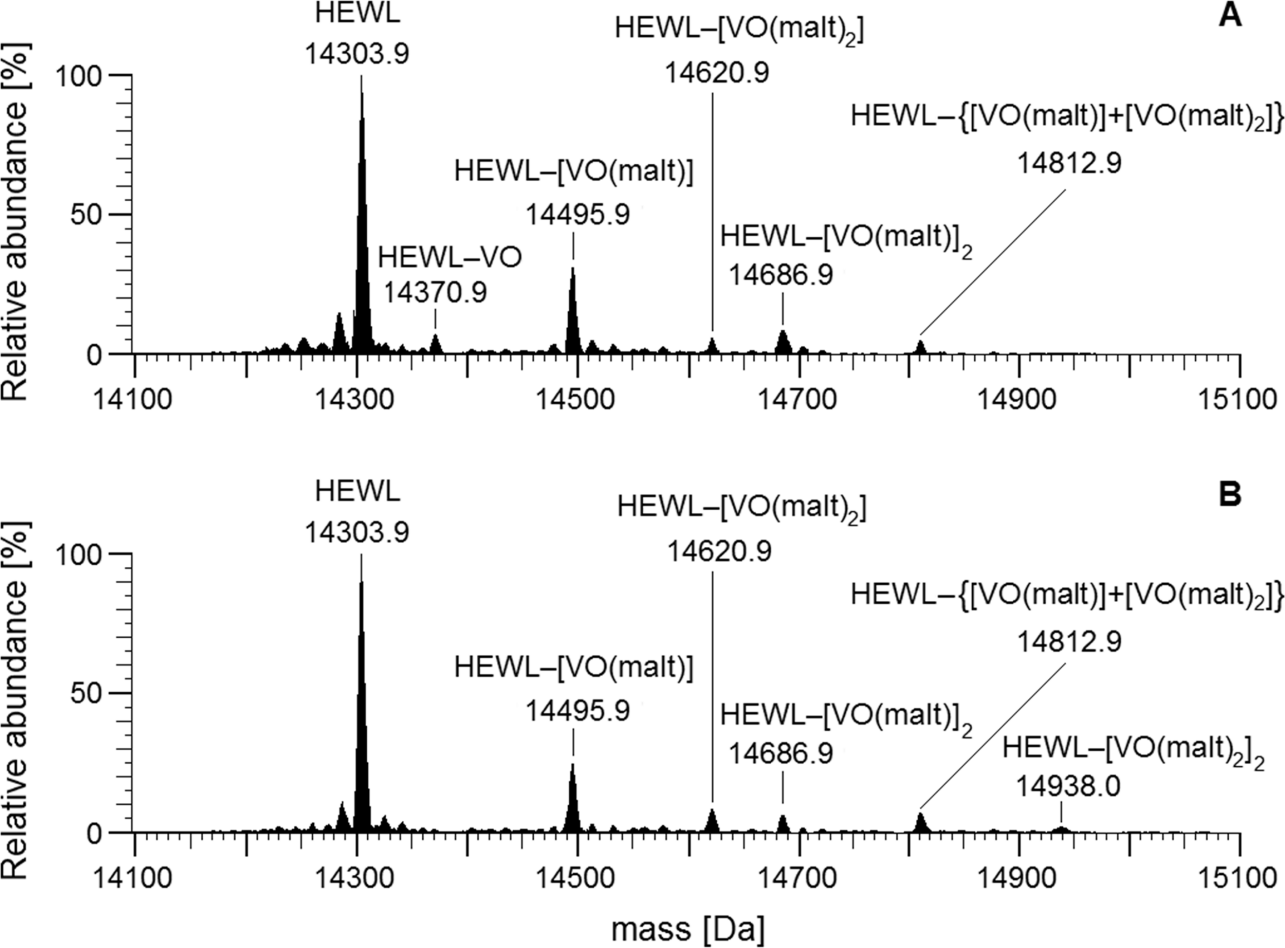
Deconvoluted ESI-MS(+) spectra recorded on the system containing BMOV and HEWL with a metal to protein molar ratio of 2/1 and a V concentration of 100 μM: (A) pH 5.0 and (B) pH 6.5.

To evaluate if the interaction of BMOV with HEWL takes place and establish the type and number of the possible adducts, ESI-MS(+) spectra were recorded at pH 5.0 and 6.5 with a molar ratio of 2/1 and V concentration of 100 μM. The reference raw spectrum of free HEWL shows a series of peaks with different charged states from +8 to +12 in the *m*/*z* range 1800–1200 (Figure S3A). In the deconvoluted spectrum, the central peak found at 14303.9 Da is surrounded by those of the adducts formed with Na^+^ ions or H_2_O molecules (Figure S3B). When BMOV is present in aqueous solution, in the raw spectrum, for each HEWL peak, other signals at higher *m*/*z* values are detected, indicating the formation of HEWL–VO–malt adducts, in agreement with the literature data.^17^ However, the charge state distribution does not vary appreciably upon the V-containing fragments, suggesting that HEWL maintains its folded conformation. The deconvoluted spectra recorded at pH 5.0 and 6.5 (Figure 2) give important insights: (i) at both the pH values, the interaction with V^IV^O(malt)^+^ and V^IV^O(malt)_2_ is revealed and the multiple binding of these two moieties is observed; (ii) at pH 5.0, the binding of V^IV^O^2+^ is also detected; (iii) by increasing the pH to 6.5, the number of adducts with the V^IV^O(malt)_2_ fragment (HEWL–[VO(malt)_2_] and HEWL–[VO(malt)_2_]_2_) increases. These experimental findings agree well with the results predicted through the thermodynamic stability constants^9^ and with the distribution curves of the V^IV^O species shown in Figure S2, which suggest that, at acidic pH values, the aqua ion and 1:1 species exist and the amount of 1:2 complex is limited, while going toward the neutrality, [V^IV^O(H_2_O)_4_]^2+^ disappears and the relative amount of V^IV^O(malt)_2_ compared to V^IV^O(malt)^+^ increases.

The final comment concerns the oxidation state of vanadium, for which ESI-MS results suggest +IV in the timescale used for the experiments. In fact, an oxidation to V^V^ should result in the formation of adducts based on the V^V^O_2_ moiety.

**Figure 3:**
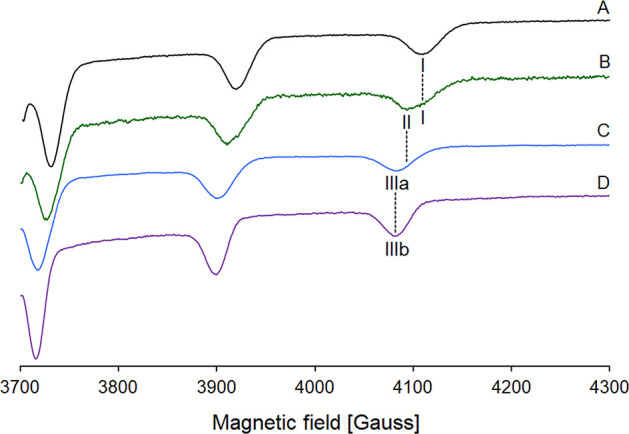
High-field region of anisotropic X-band EPR spectra recorded at pH and 120 K in the systems of an aqueous solution containing (A) [V^IV^O(malt)_2_(H_2_O)], (B) BMOV/HEWL 2/1, (C) BMOV/HSA 2/1, and (D) BMOV/MeIm 1/4. The V concentration is always 1.0 mM. The *M*_I_ = 7/2 resonance of [V^IV^O(malt)_2_(H_2_O)] is indicated with **I**, of HEWL–[V^IV^O(malt)_2_] with **II**, of *cis*-[V^IV^O(malt)_2_(HSA-His)] with **IIIa**, and of *cis*-[V^IV^O(malt)_2_(MeIm)] with **IIIb**. The coordination of the two maltolato ligands is (equatorial–equatorial) and (equatorial–axial), and the fourth equatorial donor is a water-O for **I**, an Asp/Glu-COO^–^ or an Asn/Gln-CO for **II**, and a His-N or an imidazole-N for **IIIa** and **IIIb**.

Anisotropic EPR spectra, recorded at 120 K in the system BMOV/HEWL 2/1 at pH 7.4, are shown in Figure 3. All the spectra were simulated with the software WINEPR SimFonia.^31^ The experimental and simulated signals of the full spectra are shown in Figures S4–S7, where the instrumental settings are also reported. The spin Hamiltonian parameters are listed in Table S1. The spectra were simulated assuming a tetragonal symmetry with *g_x_* = *g_y_* and *A_x_* = *A_y_*; this agrees well with the data in the literature, which suggest that the value of |*A_x_* – *A_y_*|, related to the *x*,*y* anisotropy, is very small for octahedral V^IV^O species (generally less than (2-3) × 10^–4^ cm^–1^^32^).

The spectra in the traces A, C, and D, measured at the same experimental conditions, have been added as a reference. In the spectrum of the solution containing BMOV (trace A), only the resonances of *cis*-[V^IV^O(malt)_2_(H_2_O)] are detected (denoted with **I** in Figure 3) and the hyperfine coupling constant on the *z* axis (*A_z_*) is 170.8 × 10^–4^ cm^–1^. The spectrum in trace D has been measured on the system BMOV/MeIm and is characterized by the presence of *cis*-[V^IV^O(malt)_2_(MeIm)] (**IIIb** in Figure 3), where the equatorial water is replaced by an imidazole-N, with an *A_z_* of 164.8 × 10^–4^ cm^–1^. Finally, in the spectrum reported in trace C, the resonances of the adduct of V^IV^O(malt)_2_ with human serum albumin (HSA) are observed with an *A_z_* of 165.0 × 10^–4^ cm^–1^ (**IIIa** in Figure 3); in such an adduct, the value of *A_z_* as well as the coordination around V is similar to *cis*-[V^IV^O(malt)_2_(MeIm)] (indeed, it has been denoted with HSA–[V^IV^O(malt)_2_] or *cis*-V^IV^O(malt)_2_(HSA-His) with albumin binding to V with a His-N donor).

In contrast, in the system with BMOV and HEWL, the resonances of two species (**I** and **II** in Figure 3) are observed. The resonances **I** coincide with those of *cis*-[V^IV^O(malt)_2_(H_2_O)], while the resonances indicated with **II** are intermediate between those of *cis*-[V^IV^O(malt)_2_(H_2_O)] and *cis*-[V^IV^O(malt)_2_(MeIm)]/*cis*-[V^IV^O(malt)_2_(HSA-His)] with an experimental *A_z_* of 167.8 × 10^–4^ cm^–1^ (**II** in Figure 3). This suggests that the fourth donor in the equatorial plane of the V^IV^O^2+^ ion is an O atom that—according to “additivity relationship”, the empirical rule that allows to estimate *A_z_* from the contribution of the four donors in the equatorial plane of the V^IV^O^2+^ ion^33^—should give a contribution to *A_z_* between those of an imidazole-N and a water-O. Globally, these data allowed us to assign the resonances **II** to an Asp/Glu-COO^–^ or an Asn/Gln-CO side chain and to exclude the binding of the unique histidine residue of HEWL, His15, which is the preferred donor for cisplatin and other Pt-based drugs.^34^ Compared to an equatorial water-O, the additivity relationship predicts a decrease in the value of *A_z_* of ca. (3–4) × 10^–4^ cm^–1^ upon the coordination of a carboxylate-O or carbonyl-O binding and of ca. (5–6) × 10^–4^ cm^–1^ for a His-N coordination,^33^ in agreement with what was experimentally observed.

### X-ray Study: Structures **A** and **A′**

X-ray diffraction data were collected on two crystals (structures **A** and **A′**) of HEWL grown in 2.0 M sodium formate and 0.1 M Hepes at pH 7.5 and exposed to [V^IV^O(malt)_2_] for 3 weeks. The two crystals are obtained under the same experimental conditions and using the same soaking protocol, but they come from different drops and have a different size. Crystals are isomorphous to each other and isomorphous with those of the ligand-free protein with minor differences in their unit cells (Table S2). The overall conformation of the protein in the two crystals is not significantly affected by these differences: Cα rmsd between the two structures is as low as 0.07 Å. Furthermore, it appears that the protein structure is not significantly affected by the V compound binding, confirming the ESI-MS results. Indeed, rmsd from the metal-free protein structure (PDB code 193L(26)) is 0.20 Å. Concerning the oxidation state of V, we mentioned above that the ESI-MS technique suggests the maintenance of +IV. Further experiments, evaluating the EPR intensity of the signal as a function of the time, confirm that the +IV state (3d^1^, EPR-active) is rather stable during the time range explored for the crystal preparation and manipulation (Figure S8); therefore, even if a partial oxidation to V^V^ cannot be excluded, for all the adducts, the +IV state has been considered.

**Figure 4:**
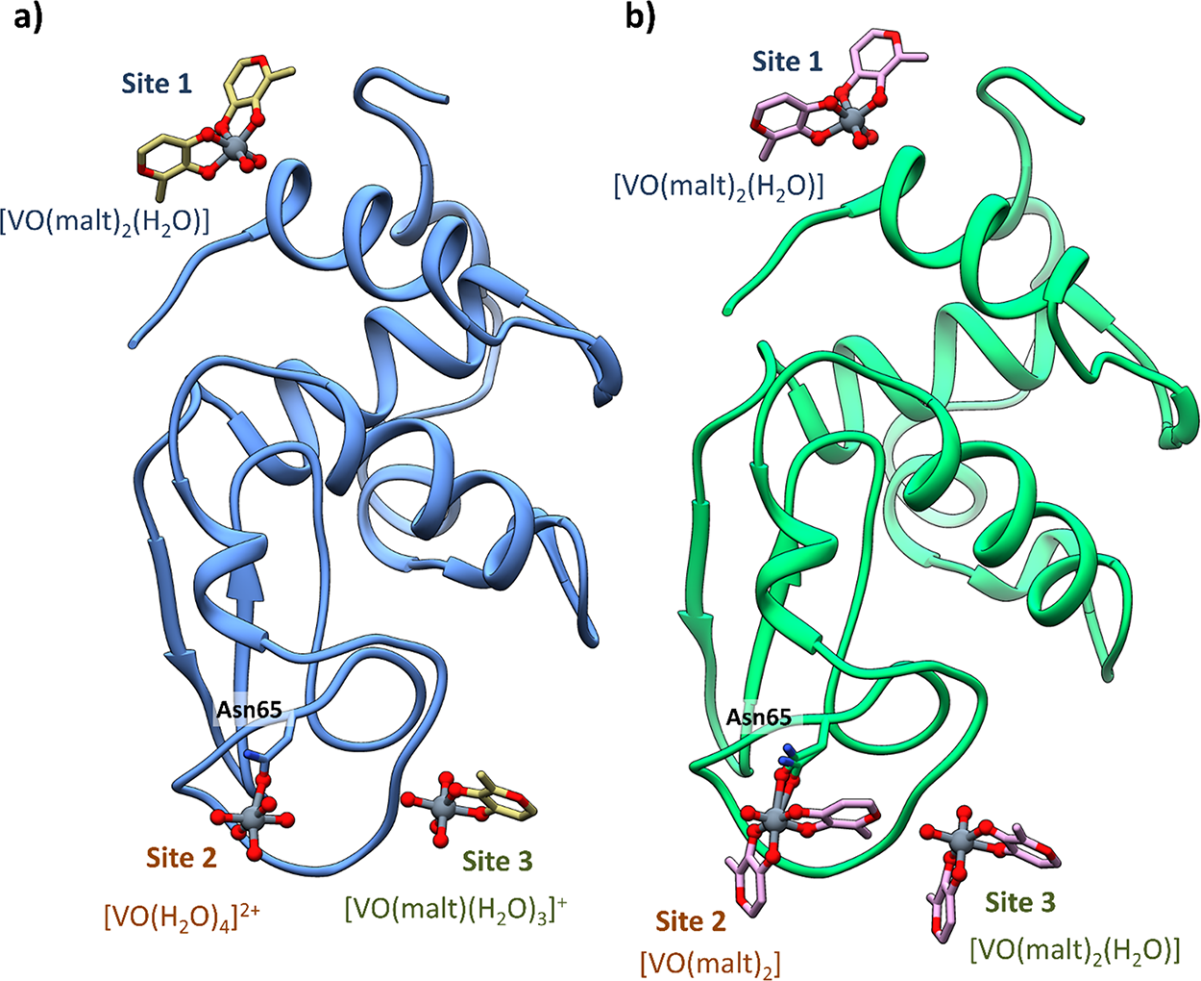
Overall structure of HEWL in the presence of [V^IV^O(malt)_2_] in 2.0 M sodium formate and 0.1 M Hepes buffer at pH 7.5 (structure **A** in panel (a) and structure **A′** in panel (b)). The position of the three binding sites **1**–**3** is also indicated.

**Figure 5:**
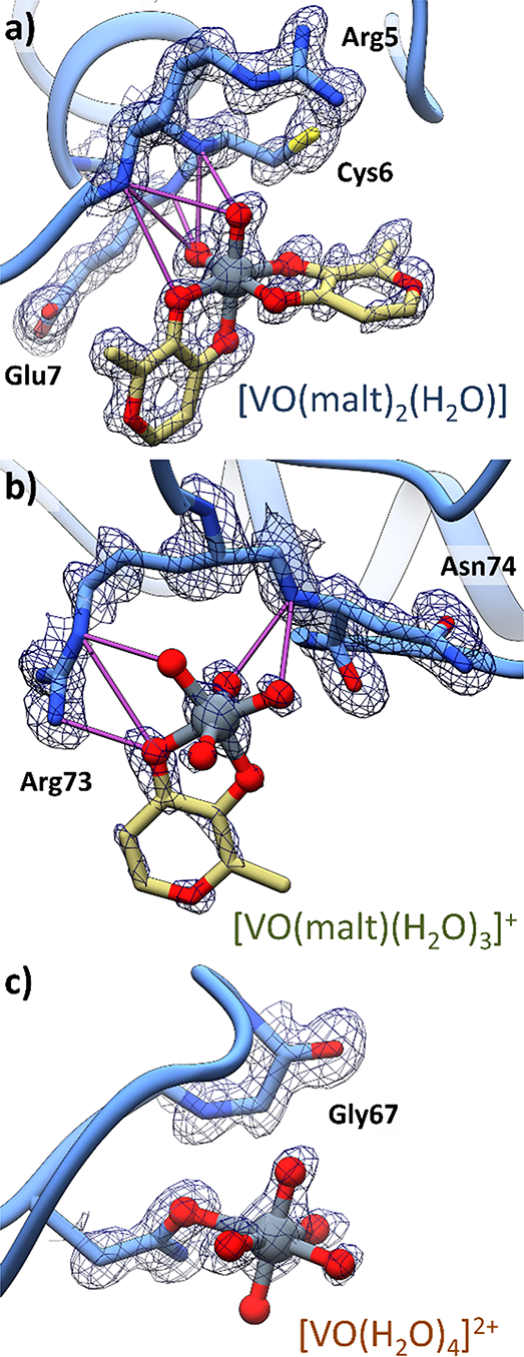
Details of the vanadium binding sites in structure **A**. Non-covalent bindings of *cis*-[VO(malt)_2_(H_2_O)] and [VO(malt)(H_2_O)_3_]^+^ to the sites **1** and **2** are reported in panels (a) and (b), respectively. The covalent binding of [VO(H_2_O)_4_]^2+^ to Asn65 of the site **3** is reported in panel (c). 2Fo-Fc electron density maps are shown at the 1.0 σ level in blue.

**Figure 6:**
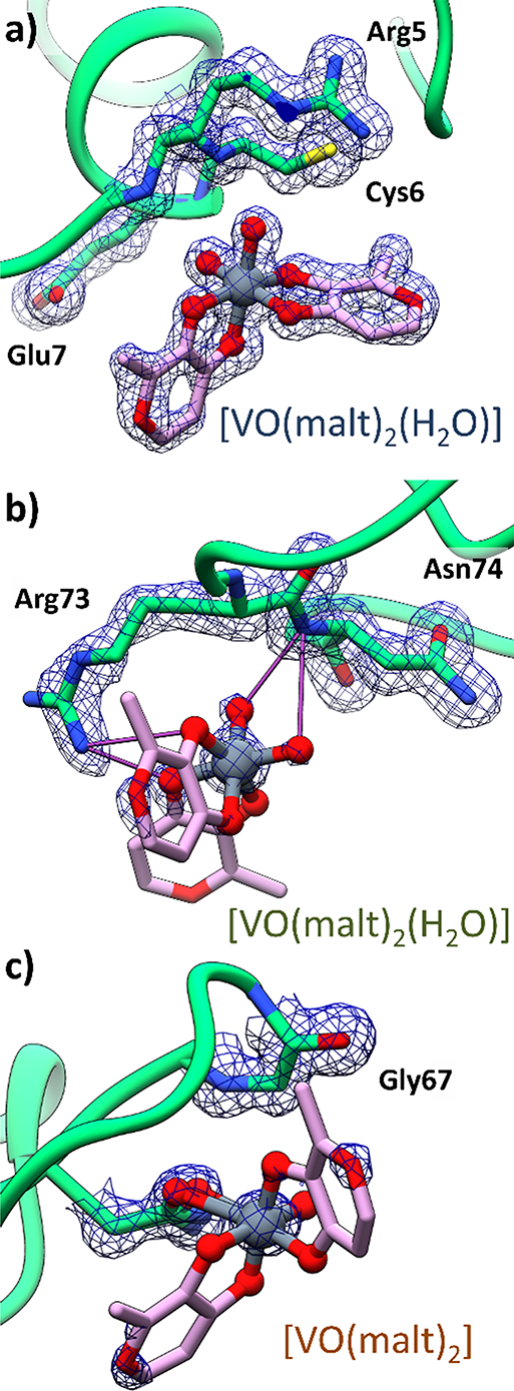
Vanadium binding sites in structure **A′**. Non-covalent bindings of the two *cis-*[VO(malt)_2_(H_2_O)] molecules to the sites **1** and **2** are reported in panels (a) and (b), respectively. The covalent binding of the [VO(malt)_2_] molecule to Asn65 of the site **3** is reported in panel (c). In this panel, the side chain of Asn65 adopts two different conformations. 2Fo-Fc electron density maps are shown at the 1.0 σ level in blue.

In both structures **A** and **A′**, three equivalent binding sites are found, named **1–3** and distinguished with light blue, orange, and green colors, respectively, in Figures 4–6. However, analysis of the diffraction data reveals significant differences between the two structures.

In structure **A**, refined at 1.13 Å resolution to R-factor/Rfree values of 0.199/0.249, respectively, non-covalent binding of *cis*-[VO(malt)_2_(H_2_O)] and [VO(malt)(H_2_O)_3_]^+^ on the protein surface was found (Figure 5a,b), together with covalent binding of a [VO(H_2_O)_4_]^2+^ ion to the side chain of Asn65 (Figure 5c). The 2Fo-Fc electron density map at these sites, reported at 1.0 σ, indicates a clear definition of the V geometry and its ligands (Figure 5a–c). The electron density is very well defined for *cis*-[VO(malt)_2_(H_2_O)] bound at the site **1** with 0.70 occupancy. For the second site (site **2**), occupied by [VO(malt)(H_2_O)_3_]^+^, the definition is a little bit low with 0.30 occupancy, while it is 0.50 for the site **3**, where [VO(H_2_O)_4_]^2+^ is bound.

In structure **A′**, non-covalent binding of two molecules of *cis*-[VO(malt)_2_(H_2_O)] (Figure 6a,b) on the protein surface and covalent binding of *cis*-[VO(malt)_2_] to the side chain of Asn65 (Figure 6c) were observed. The 2Fo-Fc electron density maps of these V binding sites are reported at 1.0 σ in Figure 6a–c. The electron density is very well defined for the first *cis*-[VO(malt)_2_(H_2_O)] (site **1**), which refines with high occupancy (0.80), while it is less defined in the case of the second *cis*-[VO(malt)_2_(H_2_O)] (site **2**) and [VO(malt)_2_] (site **3**), which have a 0.30 occupancy in the final model.

These data are in excellent agreement with the ESI-MS experiments that indicate the multiple binding of three different moieties, VO^2+^, VO(malt)^+^, and VO(malt)_2_ (see Figure 2). Moreover, the fragment VO(malt)_2_ covalently bound to HEWL in structure **A′** supports the existence in solution of the complex [V^IV^O(malt)_2_(H_2_O)], which gives the adduct HEWL–V^IV^O(malt)_2_ (revealed also by the ESI-MS technique) after the replacement of a weak water ligand by an O donor from a side chain of the Asn65 residue; in contrast, the oxidation of [V^IV^O(malt)_2_(H_2_O)] to [V^V^O_2_(malt)_2_]^−^, possible in principle, would preclude the observation of HEWL–V^V^O(malt)_2_ because the break of one of the two V^V^=O bonds by the amide-O of Asn65 is not plausible.

In both structures **A** and **A′**, site **1** (in light blue) is occupied by *cis*-[VO(malt)_2_(H_2_O)], which is hydrogen-bonded with the N atoms of the main chain of Arg5, Cys6, and Glu7 (Figures 5a and 6a), solvent water molecules, and the side chain of Arg14 of a symmetry related molecule and is in close contact with a V-containing species from a symmetry related molecule (Figure S9). At the site **2** of structure **A** (in orange), the [VO(malt)(H_2_O)_3_]^+^ ion forms hydrogen bonds with the main chain N atom of Asn74 and with the side chain of Arg73 (Figure 5b), with solvent water molecules, and with the *cis*-[VO(malt)_2_(H_2_O)] molecule from a symmetry mate (Figure S10). The site **2** of the structure **A′** is occupied by a *cis*-[VO(malt)_2_(H_2_O)] complex (Figure 6b), which forms hydrogen bonds with [VO(malt)_2_] (located at site **3**), with the side chain of Arg73, the main chain of Asn74, the *cis*-[VO(malt)_2_(H_2_O)] molecule from a symmetry mate, and water molecules; the maltolato ligand forms an additional stacking interaction with [VO(malt)_2_] from a symmetry related molecule (Figure S11). Finally, at site **3** of structure **A** (in green), a [VO(H_2_O)_4_]^2+^ cation is found coordinated to the side chain of Asn65 with the V-containing fragment held in its position by hydrogen bonds formed with the main chain of Gly67 (Figure 5c) and water molecules. In structure **A′**, in the same position, the [VO(malt)_2_] has been modeled (Figure 6c), interacting with a covalent bond to the Asn65 residue and forming hydrogen bonds with *cis*-[VO(malt)_2_(H_2_O)] and water molecules, plus additional stacking with *cis*-[VO(malt)_2_(H_2_O)] from a symmetry mate (Figure S12). Such results, which suggest the simultaneous presence of *cis*-[V^IV^O(malt)_2_(H_2_O)] (with a non-covalent bond) and HEWL–[V^IV^O(malt)_2_] (with a covalent bond) in the BMOV/HEWL system and—in addition—the covalent binding of HEWL through an O donor side chain, are perfectly in line with the EPR data (see Figure 3).

Thus, in structures **A** and **A′**, all the possible fragments derived from the transformation of BMOV in aqueous solution (Figure 1) are observed. Concerning the interaction of bis-chelated complex, the crystallographic results demonstrate that (i) the *SPY*-5 → *OC*-6 reaction takes place, as suggested by potentiometric and spectroscopic data,^8b,9^ but never supported by X-ray analysis up to now; (ii) several V-containing isomers bind, covalently or non-covalently, to the protein having two equatorial phenolato-O^–^ (site **1** of structures **A** and **A′** and site **2** of structure **A′**) or two equatorial keto-O (site **3** of structure **A′**); (iii) the interaction occurs with various enantiomers (*OC*-6-24-Λ at site **1** of both the structures, *OC*-6-24-Δ at site **2** of structure **A′**, and *OC*-6-34-Δ at site **3** of structure **A′**). These findings indicate that, in aqueous solution, the isomers and enantiomers derived by BMOV are in equilibrium and that subtle energy and steric factors, such as the hydrogen bonds, van der Waals contacts, and the chiral specificity of the protein sites, stabilize the interaction with the metal moieties as suggested by our previous reports.^13g^

### X-ray Study: Structure **B**

**Figure 7:**
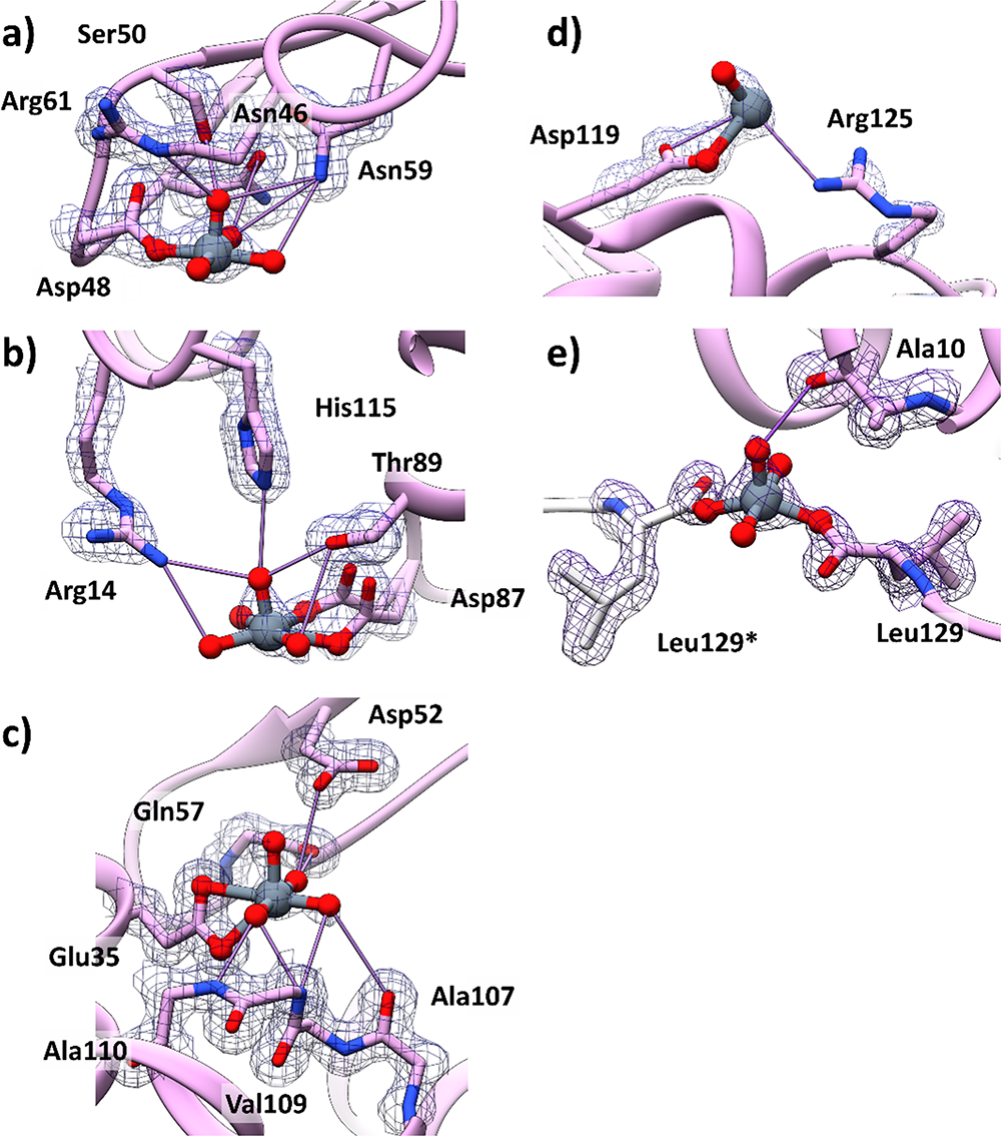
Details of the vanadium binding sites in structure **B**. Covalent binding of [VO(H_2_O)_3_]^2+^ to the side chains of Asp48 (panel a), Asp87 (panel b), and Glu35 (panel c). Other V-containing fragments are covalently bound to the side chain of Asp119 (panel d) and the C-terminal carboxylate (panel e). 2Fo-Fc electron density maps are reported at the 1.0 σ level in gray. Water molecules and nitrate ions in contact with the V ligands are omitted for the sake of clarity. Amino acid residues from symmetry related molecules are highlighted with an asterisk (*) and colored in gray.

Structure **B** has been obtained exposing HEWL crystals grown in 20% ethylene glycol, 0.1 M sodium acetate at pH 4.0, and 0.6 M sodium nitrate to a reservoir solution saturated with [V^IV^O(malt)_2_] for 3 weeks. The structure refines at 1.31 Å resolution up to R-factor and Rfree values of 0.168 and 0.198, respectively. The overall conformation of HEWL in the adduct (Figure S13) closely resembles that of the metal-free protein and of structures **A** and **A′** (rmsd within the range of 0.24 Å). In structure **B**, covalent binding of three [VO(H_2_O)_3_]^2+^ ions together with two additional V atoms, whose geometry is not well defined, is found. The first [VO(H_2_O)_3_]^2+^ ion (occupancy = 0.50) was found close to the side chain of Asp48 (Figure 7a). Here, the V atom adopts a square pyramid geometry, with the side chain of Asp48 on the plane and the V=O group at the apex of the pyramid. The V-containing fragment is held in its position by hydrogen bonds formed with the side chain of Asn46, Ser50, Asn59, and Arg61, with a water molecule and the main chain N atom of Asp48 (Figure 7a). The second [VO(H_2_O)_3_]^2+^ ion (occupancy = 0.50) was observed close to the side chain of Asp87 (Figure 7b). Here, the interpretation of the map is complicated by conformational disorder, as evidenced by the presence of alternative conformations of the side chain of Asp87 and by the presence of a nitrate ion. This V fragment is on the protein surface and in contact with the side chains of His15 and Arg14 and with water molecules. The final [VO(H_2_O)_3_]^2+^ ion was found close to the side chain of Glu35 that could coordinate V^IV^ in a bidentate fashion (Figure 7c). At this site, the electron density around the V center is not very well defined, probably also because of the low occupancy (0.35). The oxygen atoms of the V fragment interact with the main chains of Gln57, Ala107, Val109, and Ala110 and the side chain of Asp52. Notably, the residues Glu35 and Asp87 were proposed as possible candidates for V drug binding.^13k^

Furthermore, covalent binding of V atoms to the side chain of Asp119 (Figure 7d) and to the C-terminal carboxylate (Figure 7e) was observed. Close to Asp119, the V center coordinates an oxygen and could be in contact with a N atom of the side chain of Arg125, whose electron density is not well defined. At this site, although the V center presents high occupancy (1.00), inspection of the electron density map does not allow a precise definition of missing ligands. The V atom found at the C-terminal carboxylate (Leu129) is at the interface between two symmetry related molecules (Figure 7e). This V completes its coordination sphere with three water molecules. The precise identification of the V geometry in this site is complicated by the low definition of the electron density map and by the proximity of this site to the two-fold axis. The V binding induces the formation of covalently linked protein dimers, as occurs when HEWL reacts with dirhodium tetraacetate.^35^

### Comparison with Other V^IV^OL_*n*_–Protein Adducts

As pointed out in the Introduction, structural data on the interaction with proteins of V^IV^O^2+^ and its complexes V^IV^OL_*n*_, with *n* = 1–2, are still scarce.^12a^ To the best of our knowledge, only five X-ray structures have been reported in the literature: (i) HEWL–V^IV^O(pic)_2_ with the covalent binding of Asp52,^18^ (ii) RNase A–V^IV^O(pic)_2_ with the covalent binding of Glu111,^19^ (iii) trypsin–V^IV^O(pic)_2_ with the covalent binding of Ser195,^20^ and (iv) HEWL–V^IV^O(H_2_O)(bipy) and (v) HEWL–V^IV^O(H_2_O)(phen) with the simultaneous covalent binding of Asn46 and Asp52.^20^ The results obtained in this study provide further elements in the comprehension of the behavior of the systems containing V^IV^O species and proteins. Below, the most significant differences are highlighted.

First of all, the capability of binding of the Asn (and Gln) side chain has been confirmed; these donors add to the list proposed for the V^IV^O binding, namely, Asp, Glu, His, and Ser.^12^ Second, the multiple binding of V^IV^O adducts, never observed until now, has been demonstrated. Third, the binding can be covalent or non-covalent. Fourth, for a V^IV^OL_2_ complex, all the possible fragments, different for composition (V^IV^OL_2_, V^IV^OL, and V^IV^O^2+^), geometry (octahedral and square pyramidal), isomerism (*OC*-6 and *SPY*-5 species), and enantiomerism (Δ and Λ enantiomers), can interact with proteins.

Finally, it must be remembered that the possibility of oxidation of V^IV^ to V^V^, depending on the crystallization conditions and type and stability of the V^IV^OL_2_ complex, cannot be excluded.

## Conclusions

In conclusion, here, we have reported the crystal structures of the adducts formed upon reaction of the potential drug [V^IV^O(malt)_2_] (abbreviated in the literature with BMOV) with HEWL, under two different experimental conditions. An analysis of the data reveals that (i) the protein does not interact with the dissolved square pyramidal compound, [V^IV^O(malt)_2_], but with its fragments in aqueous solution, i.e., *cis*-[VO(malt)_2_(H_2_O)], [VO(malt)(H_2_O)_3_]^+^, and the aqua ion [VO(H_2_O)_4_]^2+^; (ii) the obtained fragments can bind the protein both covalently and non-covalently; (iii) HEWL contains several solvent-accessible binding sites on its surface; (iv) a different number of adducts on such sites is observed and a multiple binding of V occurs; (v) the interaction of the vanadium-containing moieties does not induce any significant structural variation of HEWL; (vi) the complex [V^IV^O(malt)_2_(H_2_O)] exists in several isomers and enantiomers, and many of them can interact with the protein, depending on the chiral specificity of the protein sites; (vii) Asp/Glu side chains with the carboxylate (Glu35, Asp48, Asp87, and Asp119 for HEWL) and Asn and, possibly, Gln with a carbonyl group (Asn65 for HEWL) are the residues mainly involved in the V binding; (viii) ESI-MS and EPR techniques allow us to support the crystallographic results, confirming themselves as very valuable tools for the study of V^IV^–protein interaction; (ix) the occupancies of V-containing fragments are often >0.5 and sometimes very close to 1. Although it is not possible to derive a direct relation between the occupancy and the affinity of the metal-containing fragment for a protein, this latter result merits attention since it is a feature not found in the protein metalation by Pt,^36^ Au,^37^ Ru,^38^ and Rh^39^ drugs.

Overall, the results of the present structural and spectrometric/spectroscopic analysis fortify the concept that a biologically active V^IV^OL_2_ compound can lose its carrier ligand L before and upon interaction with proteins, also forming derivatives with water molecules replacing the carrier ligand. The simultaneous covalent and non-covalent interactions, each realized with variable strength, allow the multiple binding of various vanadium-containing fragments and the possibility that several metal moieties are transported in bloodstream and cellular environment toward the targets in the organism, amplifying the effect of the potential drug.

Finally, the reactivity of BMOV with HEWL elucidated in this paper could help in understanding of the mechanisms at the basis of the formation of V^IV^O–(carrier L)–protein adducts that biologically active VOL_2_ compounds form with transferrin, albumin, or other membrane or cytosolic proteins, promoting and boosting the development of new V complexes as putative therapeutic agents.

## Supporting Information Available

Tables with EPR parameters (Table S1) and X-ray diffraction data collection and refinement statistics (Table S2), figures with isomers of [V^IV^O(malt)_2_(H_2_O)] (Figure S1), concentration distribution curves of the V^IV^O^2+^/malt system (Figure S2), ESI-MS spectra of HEWL (Figure S3), simulated EPR spectra (Figures S4–S7), time dependence of EPR intensity (Figure S8), and interactions of the metal fragments and HEWL in structures **A**, **A′**, and **B** (Figures S9–S13) (PDF)
