# Supplementary material for: Multiple and Variable Binding of Pharmacologically Active Bis(maltolato)oxidovanadium(IV) to Lysozyme
Source: Inorg Chem. 2022 Oct 7;61(41):16458–67. doi: 10.1021/acs.inorgchem.2c02690 (PMC9579999; doi:10.1021/acs.inorgchem.2c02690)
Supplement: Supplementary file 1 — ic2c02690_si_001.pdf [file ic2c02690_si_001.pdf]

SUPPORTING INFORMATION

Multiple and variable binding of pharmacologically active BMOV to lysozyme

Giarita Ferraro,[a] *Maddalena Paolillo,*[a] *Giuseppe Sciortino,*[c,d] *Eugenio Garribba**[c], *Antonello Merlino,**[a]

[a] Department of Chemical Sciences, University of Naples Federico II, I-80126 Napoli, Italy

[b] Institute of Chemical Research of Catalonia (ICIQ), The Barcelona Institute of Science and Technology, 43007 Tarragona, Spain

[c] Dipartimento di Medicina, Chirurgia e Farmacia, Università di Sassari, Viale San Pietro, I-07100 Sassari, Italy

Corresponding authors. E-mail: garribba@uniss.it (E.G.); antonello.merlino@unina.it (A.M.).

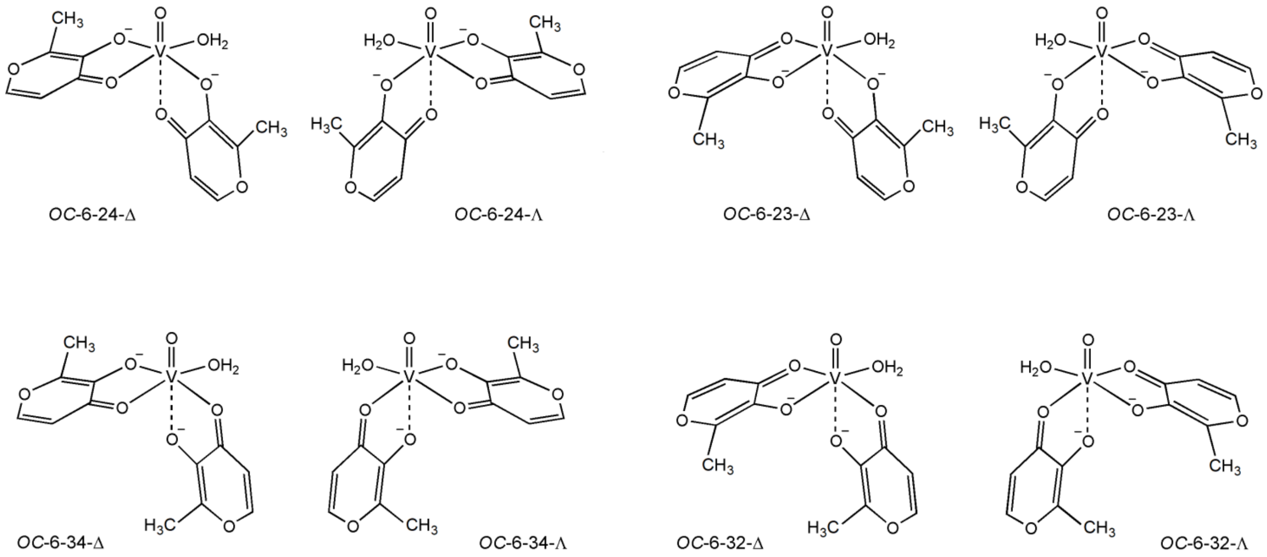

Figure S1. Isomers/enantiomers of *cis*-[$V^{IV}O(malt)_2(H_2O)$] complex formed after the dissolution of BMOV at neutral pH.

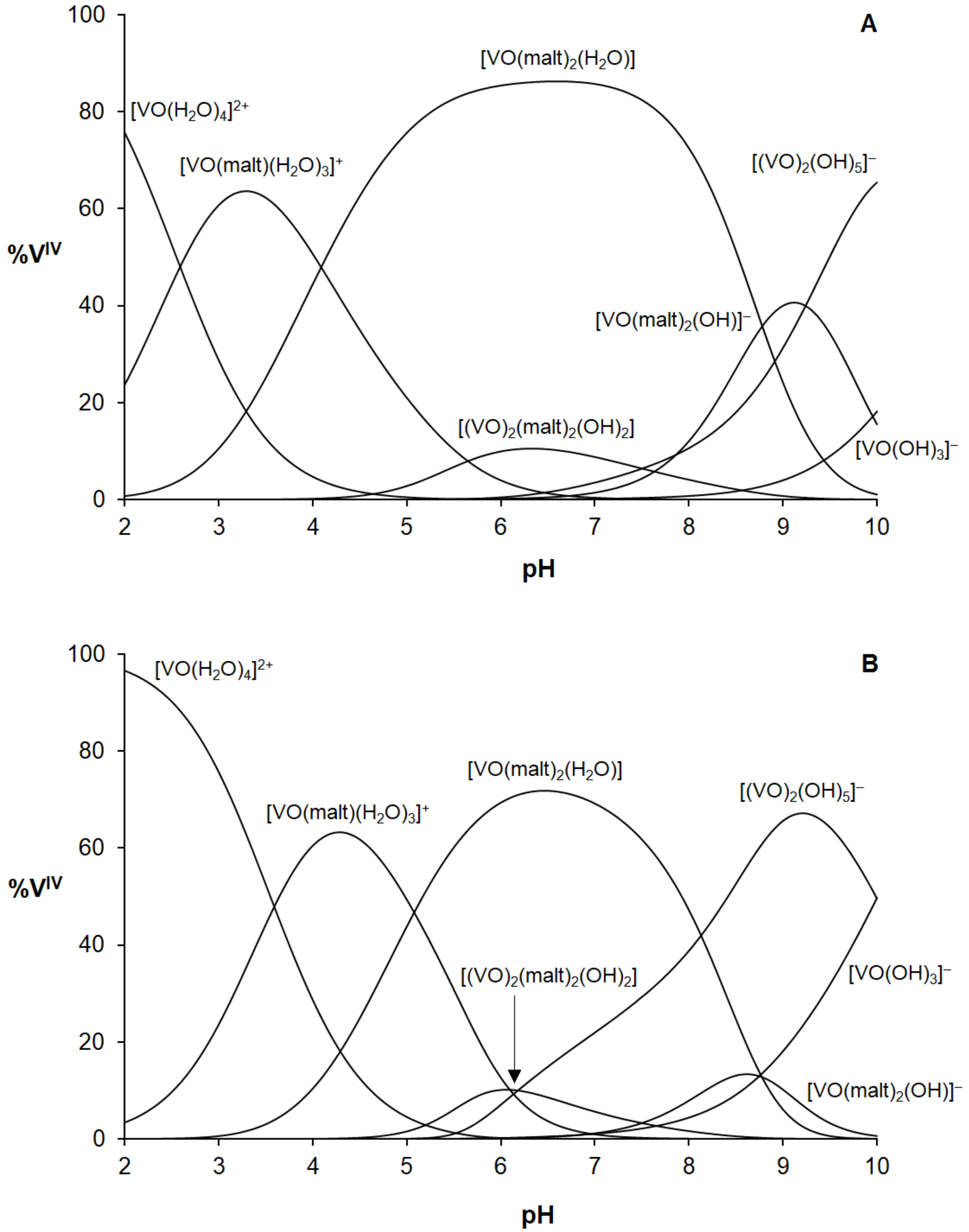

Figure S2. Concentration distribution curves of the species formed as a function of pH in the system $V^{IV}O^{2+}$/malt 1/2. A) V concentration 1 mM; B) vanadium concentration 100 μM.

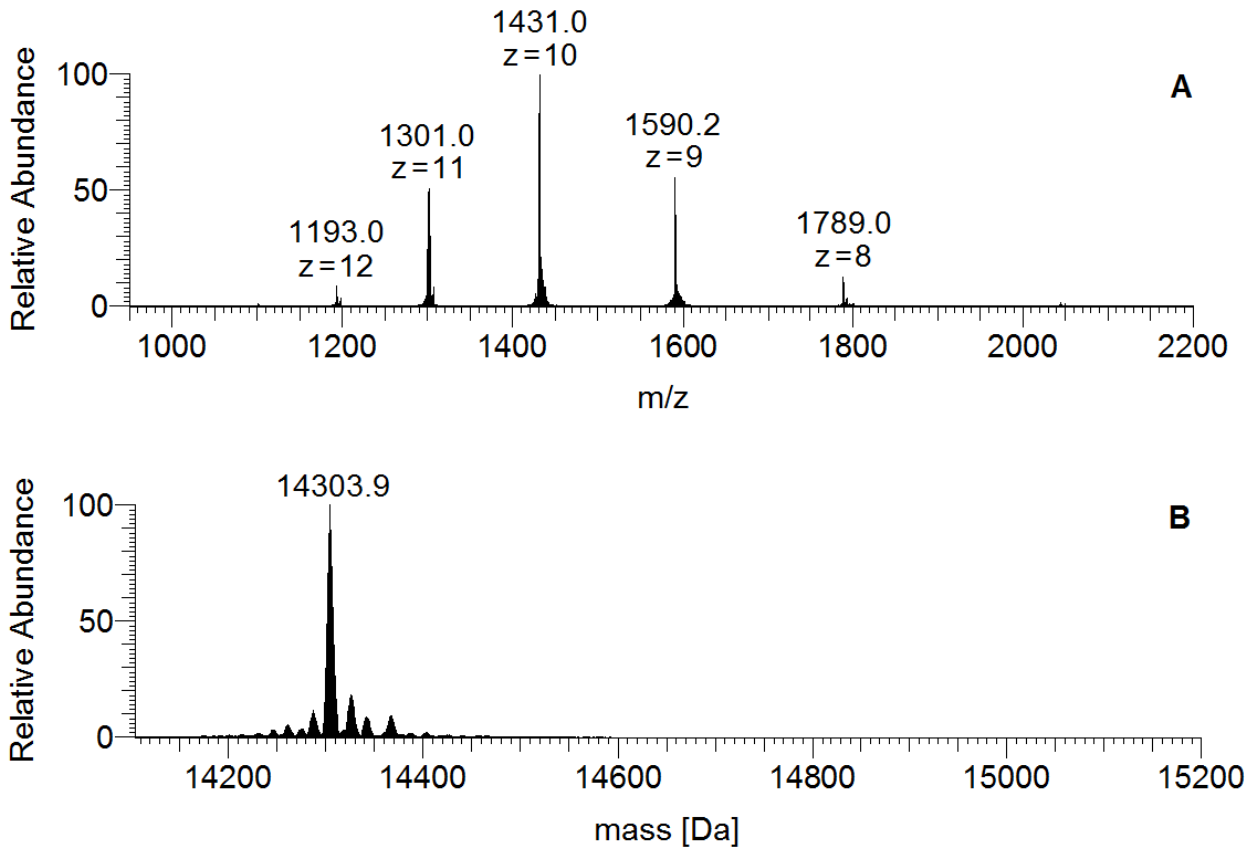

Figure S3. A) Raw and B) deconvoluted ESI-MS spectrum of HEWL (concentration 50 μM).

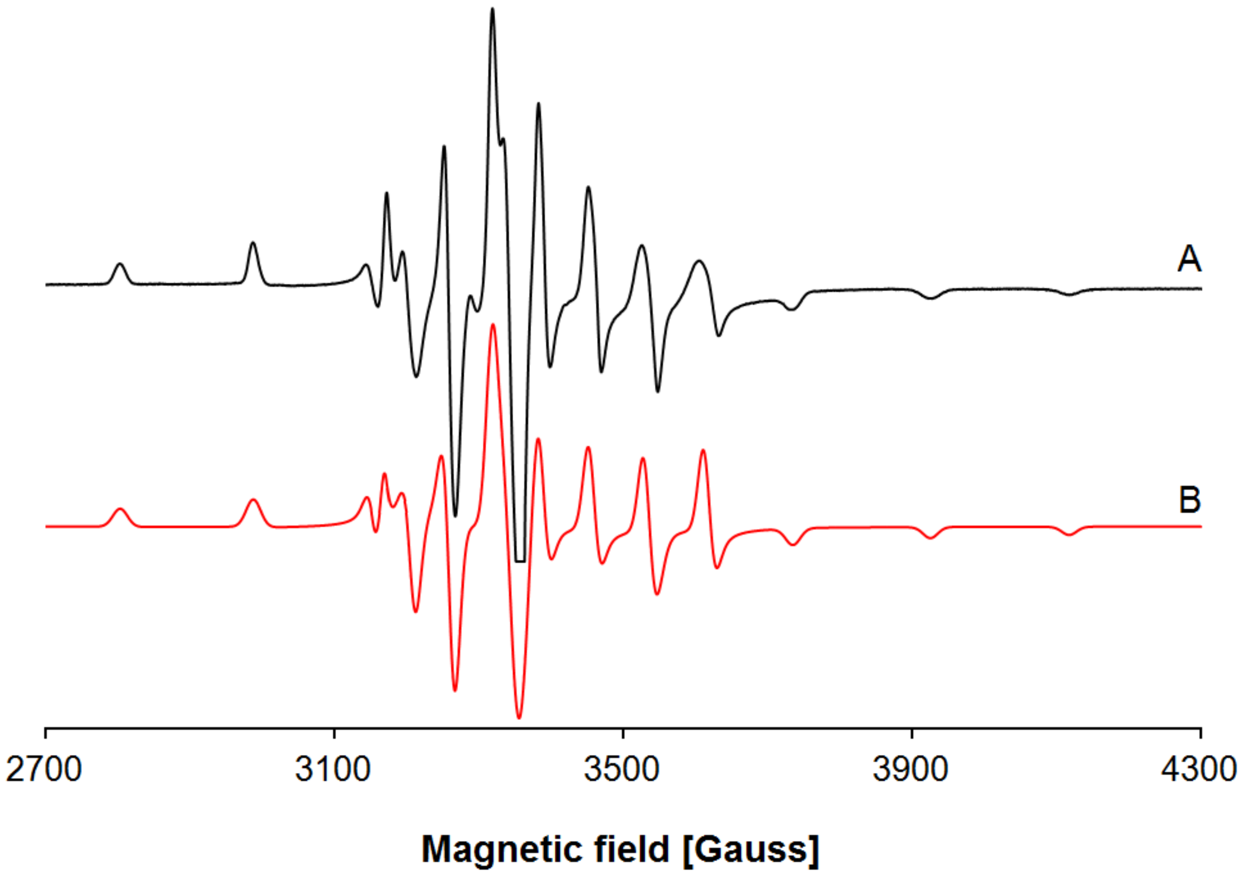

Figure S4. Experimental (in black) and simulated (in red) spectrum of $[V^{IV}O(malt)_2(H_2O)]$ with water-O equatorial binding. The instrumental parameters to record the spectrum were: microwave frequency 9.404 GHz, microwave power 20.2 mW, modulation frequency 100 kHz, modulation amplitude 4 Gauss, time constant 81.92 ms, sweep time 335.5 s, resolution 4096 points. The spin Hamiltonian parameters used for the simulation were g_x = 1.978, g_y = 1.978, g_z = 1.941, A_x = -60.4 × 10^{-4} cm^{-1}, A_y = -60.4 × 10^{-4} cm^{-1}, A_z = -170.8 × 10^{-4} cm^{-1}. The linewidth on the x, y, and z axes was 17, 17 and 20 Gauss and the Lorentzian/Gaussian ratio was 1.0. The spectrum was simulated with WinEPR SimFonia software.

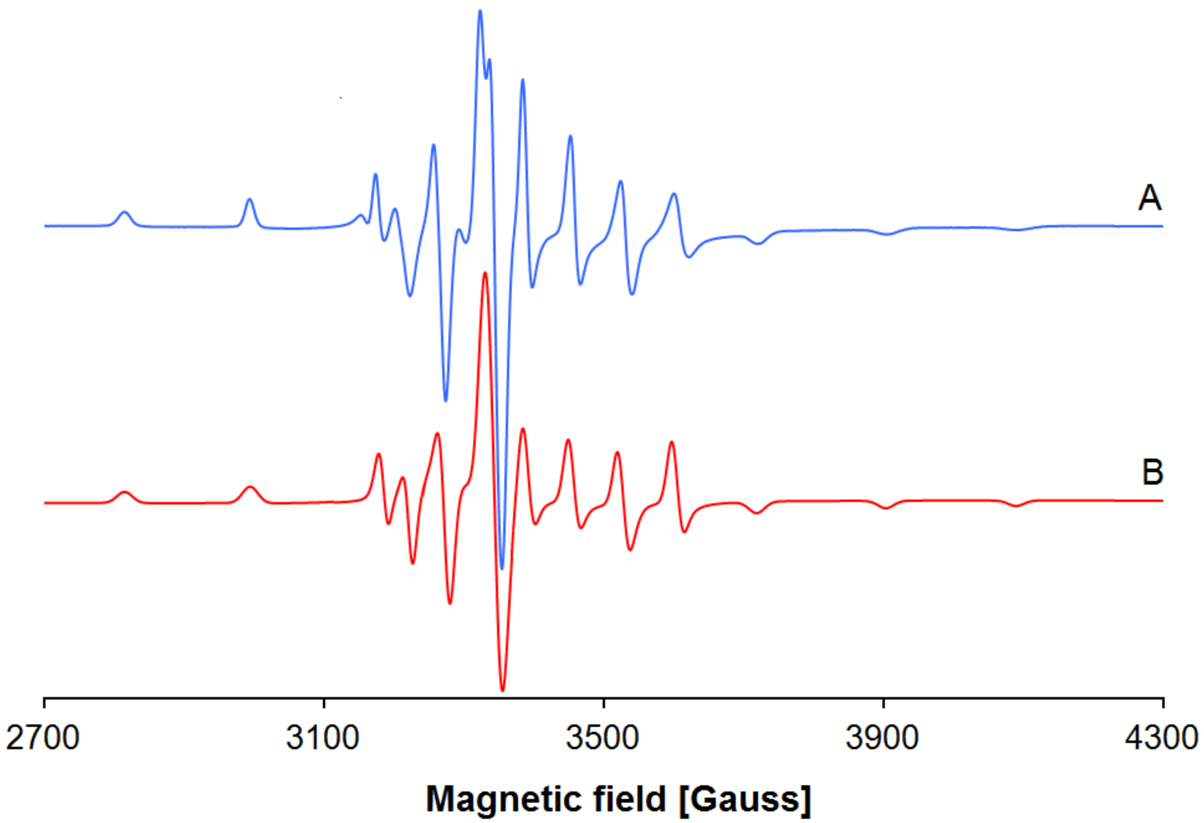

Figure S5. Experimental and simulated spectrum of the adduct HSA–[$V^{IV}O(malt)_2$] with His-N equatorial binding. The instrumental parameters to record the spectrum were: microwave frequency 9.429 GHz, microwave power 20.2 mW, modulation frequency 100 kHz, modulation amplitude 4 Gauss, time constant 81.92 ms, sweep time 335.5 s, resolution 4096 points. The spin Hamiltonian parameters used for the simulation were g_x = 1.981, g_y = 1.981, g_z = 1.950, A_x = -56.1 × 10^{-4} cm^{-1}, A_y = -56.1 × 10^{-4} cm^{-1}, A_z = -165.0 × 10^{-4} cm^{-1}. The linewidth on the x, y, and z axes was 16, 16 and 22 Gauss and the Lorentzian/Gaussian ratio was 1.0. The spectrum was simulated with WinEPR software.

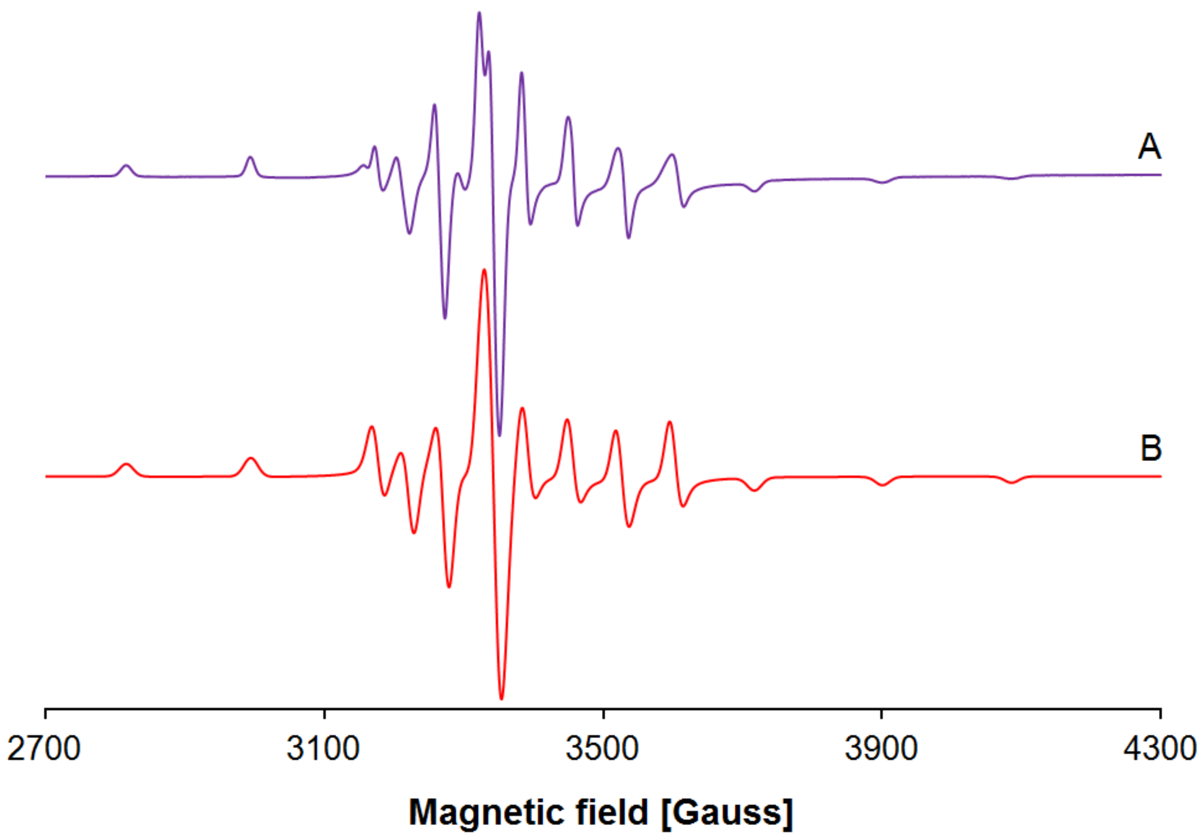

Figure S6. Experimental and simulated spectrum of the adduct [$V^{IV}O(malt)_2(MeIm)$] with imidazole-N equatorial binding. The instrumental parameters to record the spectrum were: microwave frequency 9.419 GHz, microwave power 20.2 mW, modulation frequency 100 kHz, modulation amplitude 4 Gauss, time constant 81.92 ms, sweep time 335.5 s, resolution 4096 points. The spin Hamiltonian parameters used for the simulation were g_x = 1.980, g_y = 1.980, g_z = 1.949, A_x = -55.8 × 10^{-4} cm^{-1}, A_y = -55.8 × 10^{-4} cm^{-1}, A_z = -164.8 × 10^{-4} cm^{-1}. The linewidth on the x, y, and z axes was 17, 17 and 20 Gauss and the Lorentzian/Gaussian ratio was 1.0. The spectrum was simulated with WinEPR software.

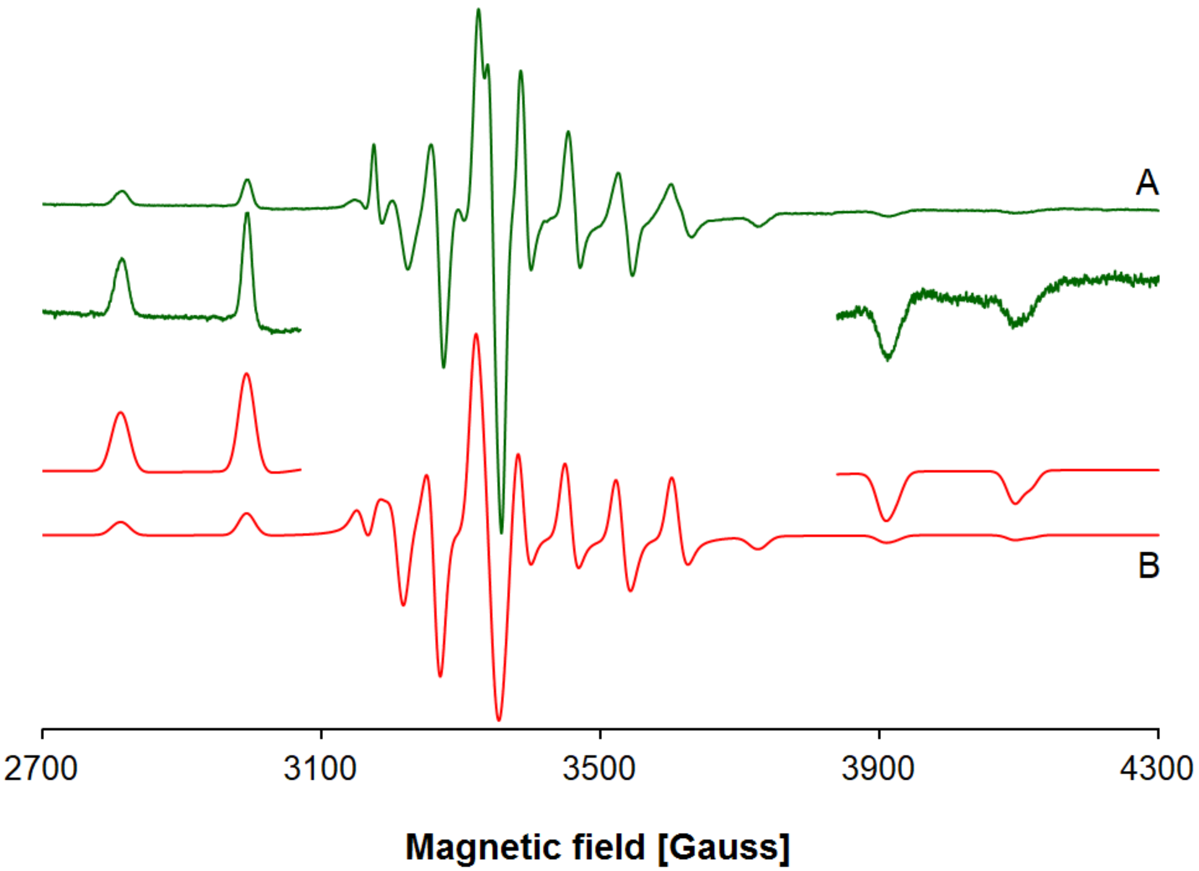

Figure S7. Experimental and simulated spectrum recorded at pH 7.4 in the system BMOV/HEWL 2/1 with vanadium concentration of 1.0 mM. The instrumental parameters to record the spectrum were: microwave frequency 9.419 GHz, microwave power 20.2 mW, modulation frequency 100 kHz, modulation amplitude 4 Gauss, time constant 81.92 ms, sweep time 335.5 s, resolution 4096 points. The spectrum was simulated overlapping the signals of two species: HEWL–[$V^{IV}O(malt)_2$] with equatorial O bonding (from Asp/Glu-COO or Asn/Glu-CO) with g_x = 1.979, g_y = 1.979, g_z = 1.944, A_x = -58.0 × 10^{-4} cm^{-1}, A_y = -58.0 × 10^{-4} cm^{-1}, A_z = -167.8 × 10^{-4} cm^{-1} (linewidth on the x, y, and z axes were 17, 17 and 22 Gauss and Lorentzian/Gaussian ratio 1.0) with a percent amount of 70% and [$V^{IV}O(malt)_2(H_2O)$] (water-O equatorial binding) with g_x = 1.978, g_y = 1.978, g_z = 1.941, A_x = -60.4 × 10^{-4} cm^{-1}, A_y = -60.4 × 10^{-4} cm^{-1}, A_z = -170.8 × 10^{-4} cm^{-1} (linewidth on the x, y, and z axes were 17, 17 and 20 Gauss and Lorentzian/Gaussian ratio 1.0) with a percent amount of 30%. The low-field and high-field regions of the two spectra have been amplified by four and ten times (trace A, experimental) and by four and six times (trace B, simulated).

Table S1. Spin Hamiltonian EPR parameters of the $V^{IV}O$ species.

Complex	g_x	g_y	g_z	A_x [a]	A_y [a]	A_z [a]
$[V^{IV}O(malt)_2(H_2O)]$ [b]	1.978	1.978	1.941	-60.4	-60.4	-170.8
HEWL–$[V^{IV}O(malt)_2]$ [c]	1.979	1.979	1.944	-58.0	-58.0	-167.8
HSA–$[V^{IV}O(malt)_2]$ [d]	1.981	1.981	1.950	-56.1	-56.1	-165.0
$[V^{IV}O(malt)_2(MeIm)]$ [e]	1.980	1.980	1.949	-55.8	-55.8	-164.8

[a] Values reported in 10^{-4} cm^{-1}. [b] Species indicated with **I** in Fig. 3 of the main text; a water-O donor occupies the fourth equatorial position [c] Species indicated with **II** in Fig. 3 of the main text; an Asp/Glu-COO^{-} or an Asn/Gln-CO donor occupies the fourth equatorial position. [d] Species indicated with **IIIa** in Fig. 3 of the main text; a His-N donor occupies the fourth equatorial position. It is also indicated with *cis*-$[V^{IV}O(malt)_2(HSA\text{-}His)]$ in the text. [e] Species indicated with **IIIb** in Fig. 3 of the main text; an imidazole-N donor occupies the fourth equatorial position.

Table S2. Data collection and refinement statistics.

	**Structure A** [a]	**Structure A'** [b]	**Structure B** [c]
Space group	$P4_3\ 2_1\ 2$	$P4_3\ 2_1\ 2$	$P4_3\ 2_1\ 2$
a (Å)	76.89	77.08	78.23
b (Å)	76.89	77.08	78.23
c (Å)	38.05	38.09	37.28
α/β/γ (°)	90.0/90.0/90.0	90.0/90.0/90.0	90.0/90.0/90.0
Molecules for asymmetric unit	1	1	1
Resolution range (Å)	54.37-1.13 (1.15- 1.13)	38.54-1.22 (1.24-1.22)	55.32-1.31 (1.33-1.31)
Observations	679735 (9265)	652273 (20573)	696797 (35107)
Unique reflections	42289 (1866)	34981 (1663)	28122 (1360)
Completeness (%)	97.3 (87.9)	99.7 (97.3)	98.9 (96.1)
Redundancy	16.1 (5.0)	18.6 (12.4)	24.8 (25.8)
Rmerge (%)	0.060 (0.339)	0.090 (1.005)	0.141 (1.847)
Average I/σ(I)	28.4 (3.4)	19.1 (3.1)	14.3 (2.2)
$CC_{1/2}$	0.999 (0.926)	0.999 (0.747)	0.997 (0.755)
Anom. completeness (%)	96.7 (80.7)	99.7 (97.1)	99.2 (97.1)
Anom. Multiplicity	8.5 (2.7)	9.9 (6.5)	13.3 (13.4)
Resolution (Å)	54.37-1.13	38.54-1.22	55.32-1.31
N° reflections	40065	33223	26662
N° reflections in working set	2762	2374	1873
Rfactor/Rfree	0.199/0.249	0.178/0.203	0.168/0.198
N° non-H atoms in the refinement	1261	1279	1281
Estimated occupancy of *cis*-$[VO(malt)_2(H_2O)]$ (1) in structure **A**	0.70		
Estimated occupancy of $[VO(malt)(H_2O)_3]^+$ (2) in structure **A**	0.30		
Estimated occupancy of V in $[VO(H_2O)_4]^{2+}$ (1) in structure **A**	0.50		
B-factor overall ($Å^2$)	16.15	17.15	18.92

B-factor of *cis*-[$VO(malt)_2(H_2O)$] (1) in structure **A** (Å^2)	14.99		
B-factor of [$VO(malt)(H_2O)_3$]$^+$ (2) in structure **A** (Å^2)	12.21		
B-factor of V in [$VO(H_2O)_4$]$^{2+}$ (1) in structure **A** (Å^2)	18.04		
Estimated occupancy of *cis*-[$VO(malt)_2(H_2O)$] (1) in structure **A'**		0.80	
Estimated occupancy of *cis*-[$VO(malt)_2(H_2O)$] (2) in structure **A'**		0.30	
Estimated occupancy of [$VO(malt)_2$] in structure **A'**		0.30	
B-factor of *cis*-[$VO(malt)_2(H_2O)$] (1) in structure **A'** (Å^2)		18.98	
B-factor of *cis*-[$VO(malt)_2(H_2O)$] (2) in structure **A'** (Å^2)		14.99	
B-factor of [$VO(malt)_2$] in structure **A'** (Å^2)		17.08	
Estimated occupancy of V in [$VO(H_2O)_3$]$^{2+}$ (1) in structure **B**			0.35
Estimated occupancy of V (2) in structure **B**			1.00
Estimated occupancy of V in [$VO(H_2O)_3$]$^{2+}$ (3) in structure **B**			0.50
Estimated occupancy of V in [$VO(H_2O)_3$]$^{2+}$ (4) in structure **B**			0.50
Estimated occupancy of V (5) in structure **B**			0.25
B-factor of V in [$VO(H_2O)_3$]$^{2+}$ (1) in structure **B** (Å^2)			29.45
B-factor of V (2) in structure **B** (Å^2)			94.60
B-factor of V in [$VO(H_2O)_3$]$^{2+}$ (3) in structure **B** (Å^2)			24.86
B-factor of V in [$VO(H_2O)_3$]$^{2+}$ (4) in structure **B** (Å^2)			47.64
B-factor of V (5) in structure **B** (Å^2)			24.83

Most favoured/ Additional allowed	112/6	106/6	102/3
Outliers	0	0	0
Rmsd bonds (Å)	1121	1171	1158
Rmsd angles (°)	1525	1023	1560

[a] Crystallization conditions: 2.0 M sodium formate, 0.1 M Hepes pH 7.5. [b] Crystallization conditions: 2.0 M sodium formate, 0.1 M Hepes pH 7.5. [c] Crystallization conditions: 20% ethylene glycol, 0.1 M sodium acetate pH 4.0, 0.6 M sodium nitrate.

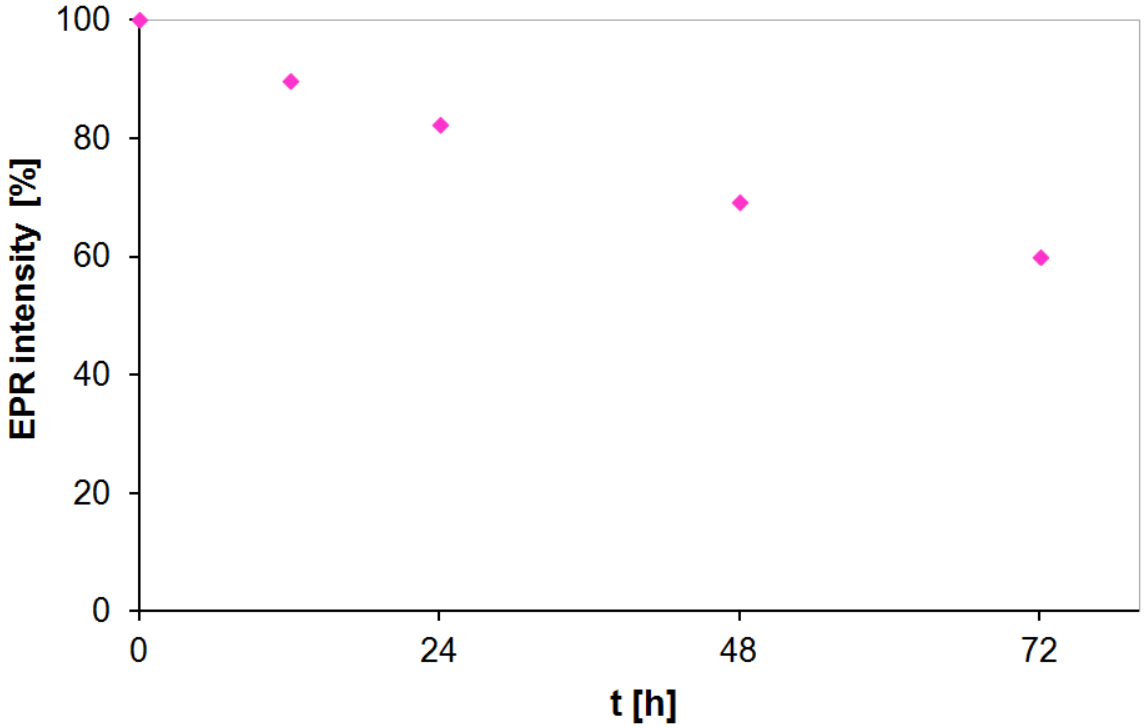

Figure S8. Time dependence of the EPR intensity (in arbitrary units) in the system BMOV/HEWL with a metal to protein molar ratio of 2/1 and a V concentration of 1 mM at pH 6.5.

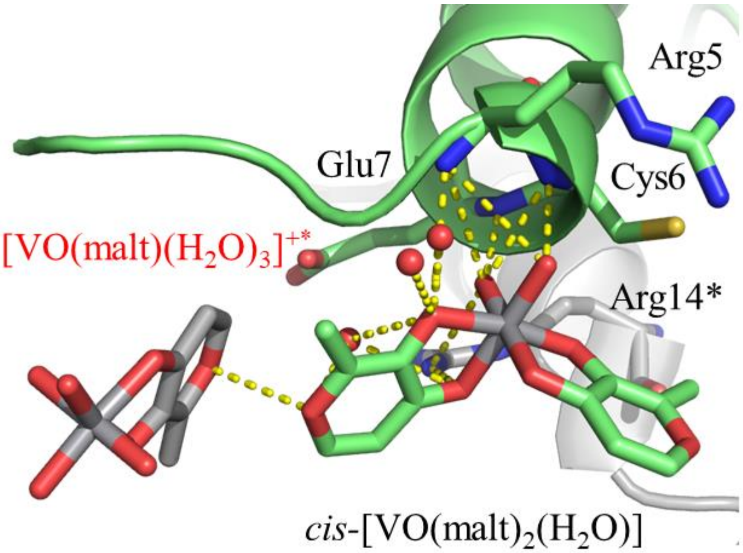

Figure S9. Interactions of *cis*-[VO(malt)$_2$(H$_2$O)] in structure **A**. Atoms from symmetry related molecules are highlighted with an asterisk (*) and colored in grey.

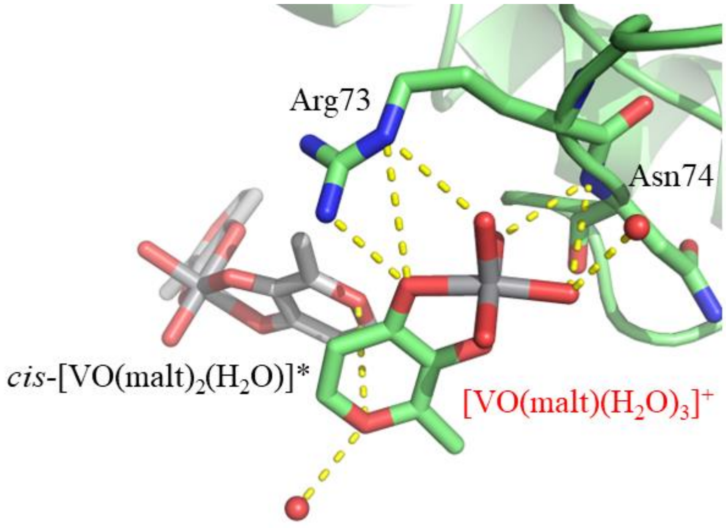

Figure S10. Interactions of $[VO(malt)(H_2O)_3]^+$ in structure **A**. Atoms from a symmetry related molecule are highlighted with an asterisk (*) and colored in grey.

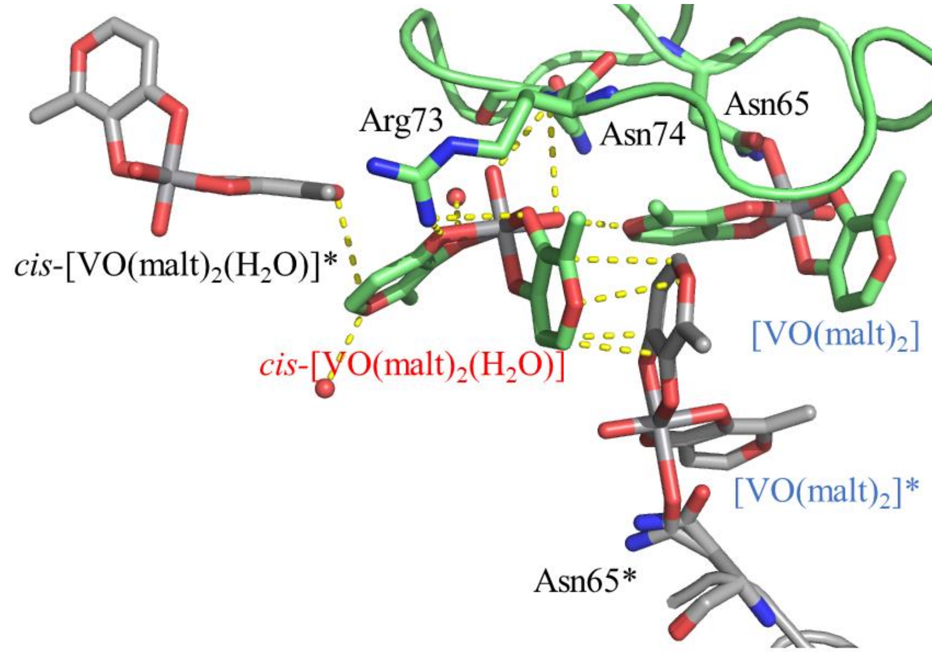

Figure S11. Interactions of the second *cis*-[VO(malt)$_2$(H_2O)] molecule in structure **A’**. Atoms from a symmetry related molecule are highlighted with an asterisk (*) and colored in grey.

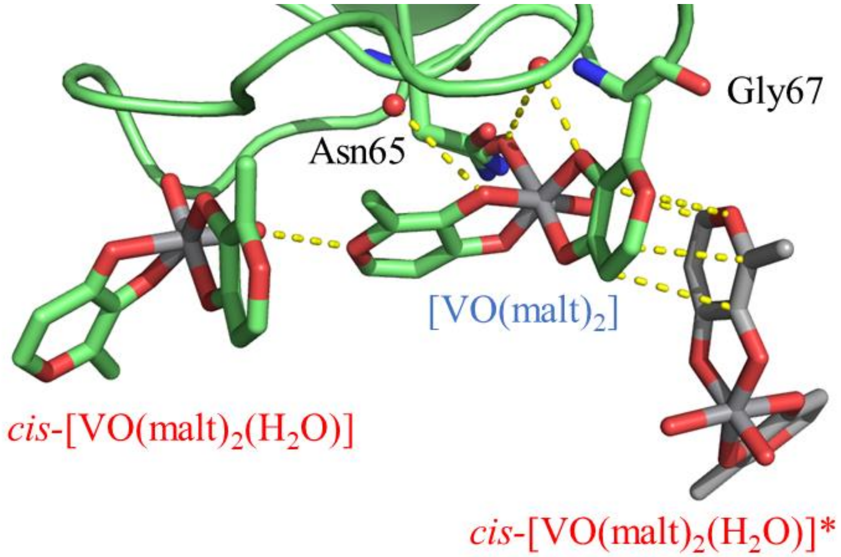

Figure S12. Interactions of [VO(malt)$_2$] molecule in structure **A'**. Atoms from a symmetry related molecule are highlighted with an asterisk (*) and colored in grey.

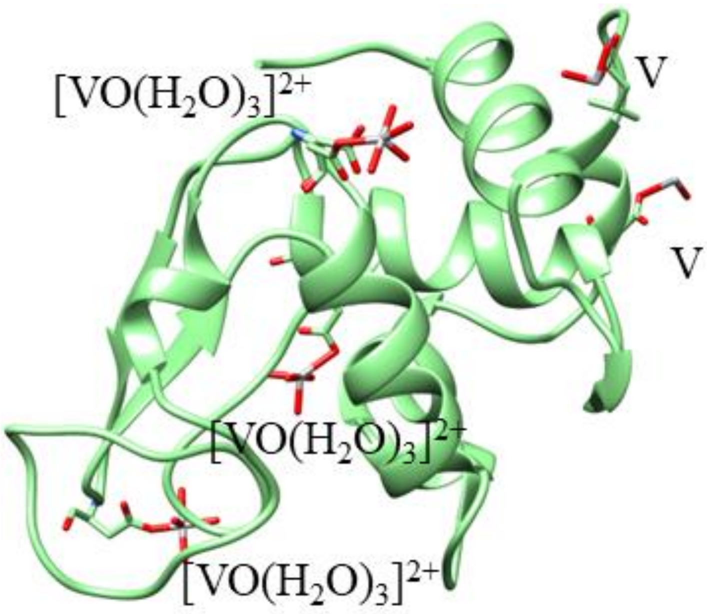

Figure S13. Overall structure of HEWL in the presence of $[V^{IV}O(malt)_2]$ in structure **B**.
